# Long-term performance of single-connector (DF4) implantable defibrillator leads

**DOI:** 10.1093/europace/euad347

**Published:** 2023-11-24

**Authors:** Rand Ibrahim, Mounir Al-Gibbawi, Wissam Mekary, Neal Kumar Bhatia, Soroosh Kiani, Stacy B Westerman, Anand D Shah, Michael S Lloyd, Miguel Leal, David B De Lurgio, Anshul M Patel, Christine Tompkins, Angel R Leon, Faisal M Merchant, Mikhael F El-Chami

**Affiliations:** Division of Cardiology, Section of Electrophysiology, Emory University School of Medicine, 550 Peachtree Street NE, 30308 Atlanta, GA, USA; Division of Cardiology, Section of Electrophysiology, Emory University School of Medicine, 550 Peachtree Street NE, 30308 Atlanta, GA, USA; Division of Cardiology, Section of Electrophysiology, Emory University School of Medicine, 550 Peachtree Street NE, 30308 Atlanta, GA, USA; Division of Cardiology, Section of Electrophysiology, Emory University School of Medicine, 550 Peachtree Street NE, 30308 Atlanta, GA, USA; Division of Cardiology, Section of Electrophysiology, Emory University School of Medicine, 550 Peachtree Street NE, 30308 Atlanta, GA, USA; Division of Cardiology, Section of Electrophysiology, Emory University School of Medicine, 550 Peachtree Street NE, 30308 Atlanta, GA, USA; Division of Cardiology, Section of Electrophysiology, Emory University School of Medicine, 550 Peachtree Street NE, 30308 Atlanta, GA, USA; Division of Cardiology, Section of Electrophysiology, Emory University School of Medicine, 550 Peachtree Street NE, 30308 Atlanta, GA, USA; Division of Cardiology, Section of Electrophysiology, Emory University School of Medicine, 550 Peachtree Street NE, 30308 Atlanta, GA, USA; Division of Cardiology, Section of Electrophysiology, Emory University School of Medicine, 550 Peachtree Street NE, 30308 Atlanta, GA, USA; Division of Cardiology, Section of Electrophysiology, Emory University School of Medicine, 550 Peachtree Street NE, 30308 Atlanta, GA, USA; Division of Cardiology, Section of Electrophysiology, Emory University School of Medicine, 550 Peachtree Street NE, 30308 Atlanta, GA, USA; Division of Cardiology, Section of Electrophysiology, Emory University School of Medicine, 550 Peachtree Street NE, 30308 Atlanta, GA, USA; Division of Cardiology, Section of Electrophysiology, Emory University School of Medicine, 550 Peachtree Street NE, 30308 Atlanta, GA, USA; Division of Cardiology, Section of Electrophysiology, Emory University School of Medicine, 550 Peachtree Street NE, 30308 Atlanta, GA, USA

**Keywords:** ICD, DF4 leads, DF1 leads, Lead failure, Lead revision

## Abstract

**Aims:**

Single-connector (DF4) defibrillator leads have become the predominantly implanted transvenous implantable cardioverter-defibrillator lead. However, data on their long-term performance are derived predominantly from manufacturer product performance reports.

**Methods and results:**

We reviewed medical records in 5289 patients with DF4 leads between 2011 and 2023 to determine the frequency of lead-related abnormalities. We defined malfunction as any single or combination of electrical abnormalities requiring revision including a sudden increase (≥2×) in stimulation threshold, a discrete jump in high-voltage impedance, or sensing of non-physiologic intervals or noise. We documented time to failure, predictors of failure, and management strategies. Mean follow-up after implant was 4.15 ± 3.6 years (median = 3.63), with 37% of leads followed for >5 years. A total of 80 (1.5%) leads demonstrated electrical abnormalities requiring revision with an average time to failure of 4 ± 2.8 years (median = 3.5). Of the leads that malfunctioned, 62/80 (78%) were extracted and replaced with a new lead and in the other 18 cases, malfunctioned DF4 leads were abandoned, and a new lead implanted. In multivariable models, younger age at implant (OR 1.03 per year; *P* < 0.001) and the presence of Abbott/St. Jude leads increased the risk of malfunction.

**Conclusion:**

DF4 defibrillator leads demonstrate excellent longevity with >98.3% of leads followed for at least 5 years still functioning normally. Younger age at implant and lead manufacturer are associated with an increased risk of DF4 lead malfunction. The differences in lead survival between manufacturers require further investigation.

## Graphical Abstract

**Graphical Abstract:**
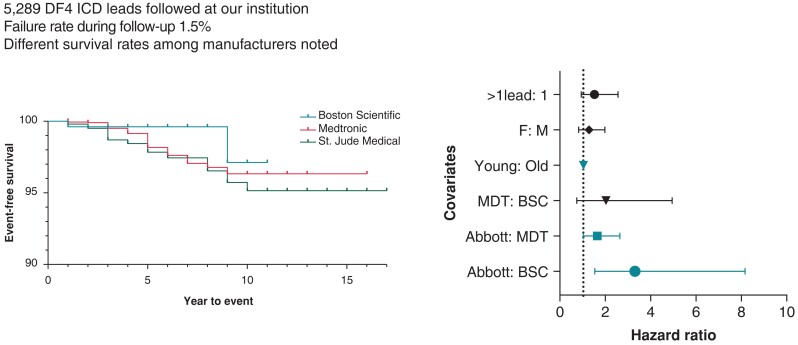

**What’s new?**

- A total of 5289 patients who underwent DF4 implantable cardioverter-defibrillator (ICD) lead implantation were followed for a mean duration of >4 years.
- DF4 ICD lead failure during follow-up was low 1.5%.
- In a multi-regression model, young age at implant and the presence of Abbott/St Jude leads were predictors of lead failure.

## Introduction

Implantable cardioverter-defibrillators (ICDs) are an established therapy for the prevention of sudden cardiac death.^1,2^ Despite the reliability of these devices, some of their components may be more predisposed to malfunction than others.^3,4^ The ICD lead, in particular, is considered the weakest link of defibrillator technology.^5^ Transvenous leads are exposed to mechanical and chemical stresses as they enter the systemic venous circulation and navigate their way to the right ventricular apex or septum. Therefore, a non-trivial and increasing rate of malfunction is seen over time. While these leads have improved in design and performance, some technological advances have resulted in unexpected and elevated malfunction rates. For instance, the urge to create small calibre leads that are less bulky and easier to implant was associated with increased malfunction rates in two commonly used ICD leads, which ultimately resulted in Class I recalls [Medtronic Sprint Fidelis and Abbott (St. Jude) Riata].^6–8^ Therefore, any new changes in lead design should be carefully monitored.^9^ Relying on manufacturer-derived product performance reports in this setting is often insufficient due to underreporting, and possibly conflicts of interest related to data provided by manufacturers.

An important change in ICD lead design was the development of DF4 leads.^10^ Some potential advantages of this new design are reduced bulk of the ICD lead in the pocket, lower potential for lead-to-can abrasion, and simplification of the implant procedure with fewer lead/header connection problems.^9^ The use of newer DF4 lead models has been on the rise globally.^11^ However, data on long-term performance of these leads are currently limited to manufacturer product performance reports and a small study with short follow-up.^12^ We sought to report on the long-term performance of DF4 ICD leads in a high-volume centre.

## Methods

Approval for this study was granted by the Emory University Institutional Review Board. Lead model numbers were collected from three major vendors supplying DF4 leads at our centre as follows: Boston Scientific (model numbers 0672, 0673, 0292, 0293, 0275, 0276), Medtronic (6935M, 6947M, 6946M), and Abbott/St. Jude (7120Q, 7121Q, 7122Q, 7170Q, 7171Q, 7172Q). A query of our institutional device clinic database (PaceArt, MURJ) was used to identify leads for inclusion. All DF4 leads evaluated at our institution were included, including those implanted by our centre as well as those implanted elsewhere but which underwent at least one interrogation at our centre. A combination of the device clinic database and electronic medical record was used to determine lead performance as well as clinical patient characteristics. The date of last device evaluation at our centre was used as the date of last follow-up.

Lead malfunction was defined as any *electrical abnormality requiring lead revision*. These abnormalities consisted of one or a combination of the following:

1. Sudden increase (≥2×) in myocardial capture threshold,
2. A sudden non-gradual change in pace-sense impedance [<200 or >2000 Ω or a significant change (≥2×) from baseline associated with another abnormality] or high-voltage impedance (<20, >120 Ω for single-coil leads, <30 and >100 for dual-coil leads),
3. Sensing of non-physiologic intervals or make/break noise excluding electromagnetic interference, and
4. Abrupt and significant change in sensing leading to under sensing.

Device interrogations and electrograms were reviewed by certified device clinic engineers and overseen by the treating cardiac electrophysiologist.

### Statistical analysis

Data analysis was conducted using the SPSS Software, version 29 and GraphPad Software, version 10. Normality testing of continuous variables was performed using the Kolmogorov–Smirnov and Shapiro–Wilk tests. This was followed by data analysis using the Student’s *t*-test or ANOVA tests. Continuous variables are reported as means with standard deviations (SDs). The χ^2^ test was used for categorical variables. Results are presented as frequencies, percentages, and odds ratios (OR) for categorical variables.

Multivariate logistic regression analysis was used to test for the effect of purposefully selected variables (age at implant, sex, presence of >1 lead) on lead malfunction. These variables were chosen based on literature suggesting their influence on lead malfunction.^13^ The variables that were found to significantly influence lead malfunction were later incorporated into Cox-regression analysis adjusting for time-to-malfunction.

Lead survival analysis was conducted by Kaplan–Meier curves using the log-rank test for stratification by manufacturer. Finally, Cox-regression models were used for adjusting baseline clinical covariates. The variables selected for Cox-regression analysis were those found to significantly affect crude survival in the preceding multivariate analysis. A 95% confidence interval (CI) is reported where applicable, and a *P*-value of ≤0.05 was considered for statistical significance.

## Results

A total of 5289 DF4 leads were evaluated at Emory healthcare from January 2011 to September 2023. Most of these leads were originally implanted and subsequently followed at our centre (*n* = 4125, 78%). Distribution across manufacturers was as follows: 1038 Boston Scientific leads, 3010 Medtronic leads, and 1241 Abbott (St. Jude) leads. The mean follow-up from implant was 4.15 ± 3.6 years (median = 3.63) with 37% of leads followed for more than 5 years.

The mean age at implant was 60.6 years [95% CI 46.3–74.8], and most patients were male (*n* = 3522, 66.6%). Baseline ejection fraction at time of implant was depressed (23.1 ± 2.2%), and most of the ICDs were implanted for primary prevention of sudden cardiac death (*n* = 3649, 69.0%). The prevalence of comorbidities was as follows: 3692 (69.8%) had hypertension (HTN), 2565 (48.5%) had diabetes mellitus (DM), 3771 (71.3%) had coronary artery disease (CAD), and 2433 (46.0%) had atrial fibrillation (AF). Baseline demographics are displayed in *Table 1*.

**Table 1:** Demographics

	*n* (5289)	%
Male	3522	66.6%
Primary indication for ICD	3649	69.0%
Hypertension (HTN)	3692	69.8%
Diabetes mellitus (DM)	2565	48.5%
Coronary artery disease (CAD)	3771	71.3%
Atrial fibrillation (AF)	2433	46.0%
	**Mean**	**Standard deviation**
Age (years)	60.6	14.2
Ejection fraction (EF, %)	23.1	2.2
Total number of implanted leads	2.03	0.9

ICD, implantable cardioverter-defibrillator.

A total of 80 leads (1.5%) experienced malfunction according to our predetermined definition. The average time to lead malfunction was 4 ± 2.8 years (median = 3.5). Lead malfunction presented with the following electrical manifestations as detailed in *Table 2*:

1. Electrical noise (*n* = 22, 28%),
2. Abnormal impedance (*n* = 22, 28%): 9 with elevated high-voltage impedance, 6 with elevated pacing impedance, 6 with low pace-sense impedance, and 1 with highly variable impedance,
3. Abrupt and significant change in sensing (*n* = 8, 10%): 8 with under sensing of ventricular electrograms,
4. Failure to capture/elevated capture thresholds (*n* = 8, 10%), and
5. A combination of the above.

**Table 2:** Detected electrical abnormalities

Electrical abnormalities	*n* (80)	%
Noise	22	28
Impedance		
High-voltage impedance	9	11
High pacing impedance	6	8
Low impedance	6	8
Variable impedances	1	1
Under sensing	8	10
Loss of capture	8	10
High DFT + loss of capture	4	5
Insulation defect (low pacing impedance or low pacing impedance + noise)	6	8
Elevated threshold	3	4
Combinations		
High pacing impedance + noise	2	3
High pacing impedance + capture failure	3	4
Noise + capture failure	2	3
	**80**	**100**

Bold values indicates total number. DFT, defibrillation threshold testing.

Lead malfunction was discovered during routine device follow-up in most cases (*n* = 48, 60%). In the remaining cases, lead malfunction came to clinical presentation with inappropriate shocks (*n* = 18, 22%), syncope (*n* = 7, 9%), heart failure exacerbation (*n* = 4, 5%) and device alerts (*n* = 3, 4%).

Failed leads were managed with transvenous lead explant/extraction in 62 cases (78%) with implantation of a new lead and in the remaining cases (*n* = 18, 22%), the malfunctioned lead was abandoned, and a new lead implanted.

**Figure 1:**
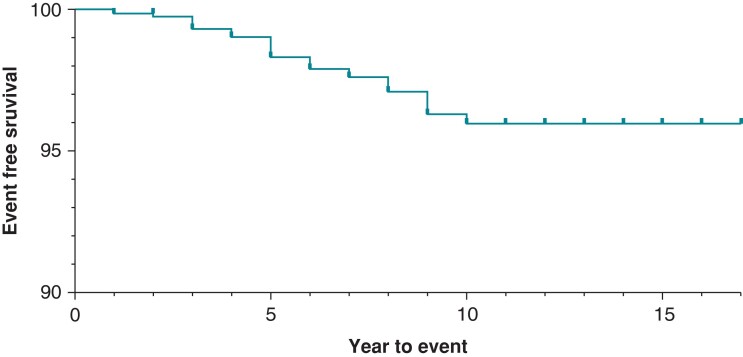
Kaplan–Meier for all leads (unstratified).

**Figure 2:**
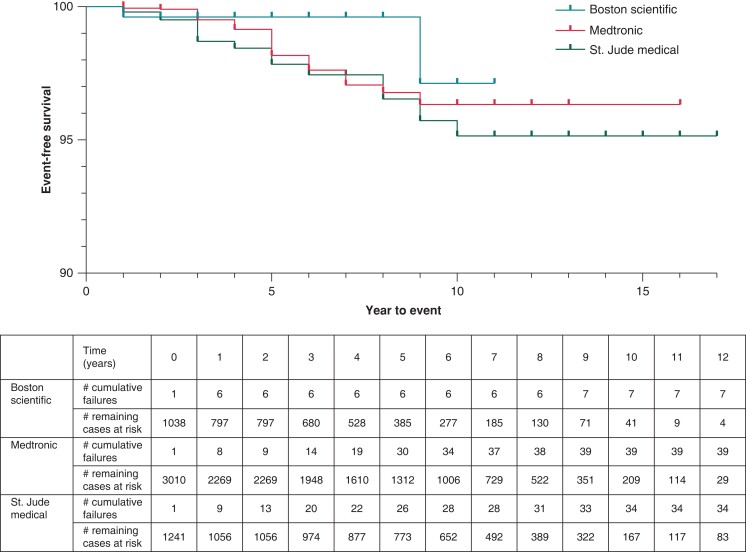
Kaplan–Meier by manufacturer (stratified).

DF4 leads in our cohort demonstrated a malfunction-free survival rate of 99.3% at 3 years of follow-up. This dropped to 98.3% for leads followed for at least 5 years. Malfunction-free survival is presented for all leads in *Figure 1*. In unadjusted analysis, lead survival was similar across manufacturers (*Figure 2*, log-rank *P* = 0.1161).

In multivariate analysis, younger age at lead implant was significantly associated with an increased risk of lead malfunction (mean implant age in malfunctioned leads of 54 ± 16 years vs. 61 ± 14 years in non-malfunction leads, *P* = 0.037). Overall, crude lead malfunction was significantly influenced by manufacturer type (*P* < 0.01). Specifically, Abbott/St. Jude was 4.2 times more likely to experience malfunction compared to Boston Scientific leads (34/1241 vs. 7/1038, *P* < 0.001). Additionally, Abbott/St. Jude leads were 2.2 times more likely to experience malfunction compared to Medtronic counterparts (34/1241 vs. 39/3010, *P* < 0.001). Finally, Medtronic leads had a trend towards higher malfunction rate compared to Boston Scientific counterparts (39/3010 vs. 7/1038, *P* = 0.054) (*Table 3*). Gender and the presence of additional leads (i.e. dual chamber or cardiac resynchronization systems) did not significantly influence lead malfunction.

**Table 3:** Multivariate regression analysis of different variables and lead malfunction

		OR	*P*-value
Younger age at implant		1.03	<0.0001
Female		1.3	0.3
Presence of >1 lead		1.6	0.15
Manufacturer	Abbott: BSc	4.2	<0.001
	Abbott: Medtronic	2.2	<0.001
	Medtronic: BSc	1.9	0.054

**Figure 3:**
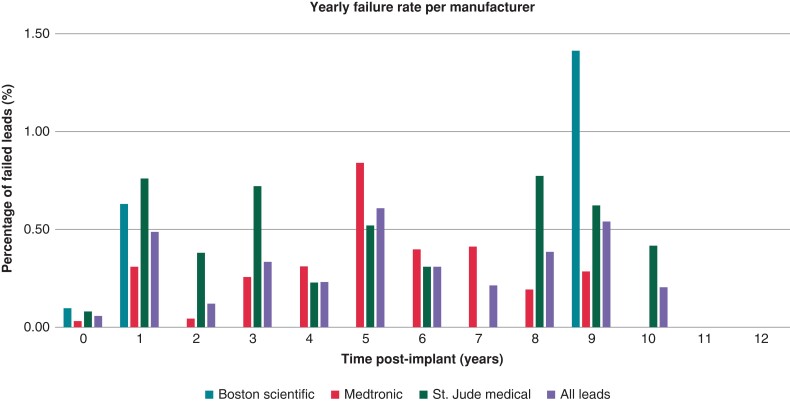
Annual rates of malfunction per manufacturer.

The annual rate of lead failure is presented in *Figure 3*. The Boston Scientific lead failure occurred at Year 1 then no failure was noted until Year 9. Medtronic leads yearly failure rate peaks at 5 years at ∼0.84%. St. Jude/Abbott leads have fluctuating yearly failure rate with peaks at Years 3 and 8.

**Figure 4:**
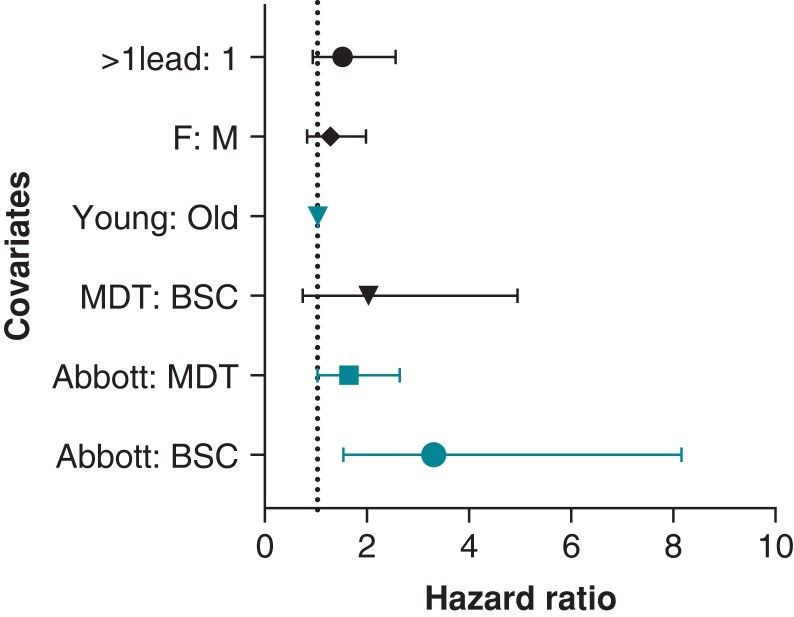
Forest plot of Cox-regression analysis.

Cox-regression models adjusted for age at implant and manufacturer revealed difference in malfunction rate across manufacturers. Medtronic leads demonstrated a trend towards higher malfunction rates compared to Boston Scientific (OR = 2, *P* = 0.086). Abbott/St. Jude leads were 1.6 and 3.3 times more likely to fail compared to Medtronic leads (*P* = 0.043) and Boston Scientific leads (*P* = 0.0046), respectively. Additionally, young age at implant was a predictor of malfunction with a malfunction rate 1.03 times higher for every year younger the patient was at implant (*P* < 0.001). There was no difference in malfunction rate between males and females (*P* = 0.3) or with the presence of additional leads (*P* = 0.11) (*Figure 4*).

## Discussion

In this single-centre study, we report on the intermediate to long-term performance of DF4 ICD leads in a large cohort of patients. In a study by Kleeman *et al.* evaluating the performance of DF1 ICD leads, the rate of malfunction was 15% during follow-up. The estimated lead survival rate was 85% at 5 years and 60% at 8 years.

In the above-mentioned study, lead failure increased with time with an annual malfunction rate of 20% at 10 years. In our study, the rate of lead failure was very low 1.5%. Also, the annual rate of failure peaked in Year 5 at 0.61% and again in Year 9 with a failure rate of 0.54%.

It is interesting to note that Boston Scientific DF4 leads had a lower rate of malfunction and plateaued early within 1 year of implant at <1%. Abbott/St. Jude leads were 4.2 times more likely to fail as compared to Boston Scientific leads and 2.2 times as compared to Medtronic leads. There were higher odds of Medtronic leads failing compared to Boston Scientific leads, but the difference was not statistically significant (*P* = 0.054). The Kaplan Meier curves diverged early and continued to separate with time (*Figures 2* and *3*) suggesting that these differences are most likely reflective of structural defects in these leads and not explained by chance.

Based on manufacturer product performance reports: Abbott/St. Jude Durata and Optisure DF4 ICD lead families have a 5-year survival rate of 96% to 98.4%. Also, Medtronic Sprint Quattro DF4 leads have a 98% survival rate at 5 years and 94% at 14 years; while the BSC ENDOTAK RELIANCE-4 leads had reportedly a 7-year survival rate of >99%. These data from the manufacturer’s product performance reports are comparable to our findings and are reassuring overall.

In the study by Kleeman *et al.*, insulation failure accounted for 56% of all causes of lead malfunction. In leads older than 6 years, insulation failure accounted for 70% of all causes of malfunction.

Abnormal impedance and noise accounted for most electrical abnormalities noted in failed leads in our study (66%) indicating that insulation failure is also responsible for a significant percentage of DF4 lead malfunction. There were no differences in incidence of low pacing impedance, noise, and insulation defect between manufacturers (Table 4). A total of 38% of lead failures in Abbott were caused by insulation failure whereas 50% of Medtronic and Boston Scientific leads failed due to insulation issues (*P* = 0.15). Optim insulation has been implicated as a possible cause of premature lead malfunction in Abbott leads, but our study results do not align with this hypothesis.^14,15^

**Table 4:** Distribution of impedance failure across manufacturers

	Boston Scientific (*n* = 7)	Medtronic (*n* = 39)	Abbott (*n* = 34)	*P*-value
Low pacing impedance	0	4	2	0.32
Insulation defect	2	1	3	0.35
Noise	1	15	8	0.14
All causes of insulation failure	3	20	13	0.15

It is not clear why the rate of DF4 lead failure is overall lower than DF1 lead failure. The strict definition of lead failure that we used in our study (any abnormality requiring lead revision) could be partly responsible for this low rate. It is also important to consider that reducing ICD lead bulk in the pocket (DF4 lead as compared to DF1 lead) could decrease the rate of insulation breech by reducing lead-to-can or lead-to-lead interaction and hence abrasion.

## Limitations

This is a single-centre retrospective study with a mean follow-up duration of 4.15 years with only 37% of leads followed for more than 5 years. Patient drop-out during follow-up could have occurred that could have affected data accuracy. Also, leads from the three biggest manufacturers were used and hence no data on lead performance from the remainder manufacturers existed. However, this was a large study examining the performance of >5000 leads. Typically, most patients with cardiac implantable electronic devices are compliant with their device follow-up and in the era of remote monitoring data from their device interrogation is documented at least once a year.

Also, our definition of lead malfunction was strict and required a decision by implanting physicians to revise these leads. It is possible that some electrical abnormalities were managed conservatively hence we could have under-appreciated the percentage of lead malfunction.

Some abnormalities such as capture threshold or sensing abnormalities that we attributed to a structural lead failure could be due to an abnormality of the lead-myocardium interface. Abnormalities of sensing and capture thresholds that are due lead-myocardium interface typically start mild and gradually worsen overtime. We only included sudden and significant changes in these electrical properties that are more likely to be due to a structural lead failure. Similarly, elevated pacing impedance could be observed in normally functioning ICD leads. However, our lead malfunction definition used a sudden and non-gradual increase in pacing impedance and all of the abnormalities were seen beyond 30 days from the implantation procedure that makes lead malfunction more likely.^16^

## Conclusions

DF4 ICD leads overall exhibit good longevity like what is reported in the manufacturer product performance report. The yearly malfunction rate remains low peaking at around Year 5 and remained <1%. Our data also suggest that there are significant differences in lead longevity between manufacturers that deserve further investigation.

## Data Availability

Data cannot be shared publicly due to privacy/ethical reasons but can be available upon reasonable request.
